# The Role of Endoscopic Ultrasound in Assessing Portal Hypertension: A State‐of‐the‐Art Literature Review and Evolving Perspectives

**DOI:** 10.1111/liv.16176

**Published:** 2024-11-27

**Authors:** Fabrizio Termite, Federica Borrelli de Andreis, Antonio Liguori, Antonio Gasbarrini, Fabia Attili, Cristiano Spada, Luca Miele

**Affiliations:** ^1^ Department of Medical and Surgical Sciences, Fondazione Policlinico Universitario Agostino Gemelli IRCCS Università Cattolica del Sacro Cuore Rome Italy; ^2^ Digestive Endoscopy Unit, Fondazione Policlinico Universitario Agostino Gemelli IRCCS, Rome Italy Università Cattolica del Sacro Cuore Rome Italy; ^3^ Digestive Endoscopy Unit Ospedale Isola Tiberina – Gemelli Isola Rome Italy; ^4^ Department of Translational Medicine and Surgery, Fondazione Policlinico Universitario Agostino Gemelli IRCCS Università Cattolica Del Sacro Cuore Rome Italy

**Keywords:** chronic liver disease, endoscopic ultrasound, portal hypertension

## Abstract

**Background:**

Portal hypertension (PH) is a critical complication in patients with hepatic diseases. Its accurate evaluation is essential for early diagnosis, risk stratification, and management. Endoscopic ultrasound (EUS) has emerged as a promising diagnostic tool, offering high‐resolution imaging of the portal venous system, hepatic vasculature, and surrounding structures.

**Aims:**

This review aims at providing an overview of the evolving role of EUS in PH evaluation in patients with liver disease.

**Materials and Methods:**

A systematic search was conducted in PubMed and Google Scholar until 31 May 2024. Relevant studies were identified using keywords related to EUS and PH. Additional references were included based on expert knowledge and citation analysis. Only full‐length papers and abstracts in English were considered. Results: EUS demonstrates significant utility in PH assessment, offering high‐resolution imaging and advanced tools like contrast enhancement (CE) and shear‐wave elastography (SWE) for evaluating liver stiffness and correlating it with PH severity. EUS‐guided portal pressure gradient (PPG) measurement provides a less invasive method for evaluating PH, potentially offering a safer alternative to conventional techniques.

**Discussion:**

EUS offers unique advantages in PH assessment, enabling comprehensive evaluation in a single session. Despite its potential, limitations such as invasiveness, sedation‐related variability, and restricted availability persist. Emerging techniques require further validation in larger cohorts and standardised training.

**Conclusion:**

EUS is a valuable diagnostic tool for PH evaluation, with the potential to improve outcomes through earlier diagnosis and better stratification. Addressing its limitations through further research and standardised protocols is critical to optimize its clinical utility.

**Trial Registration:** NCT04115046, NCT05728697, NCT05097963 and NCT03155282

**Abbreviations**
APRI: AST to platelet ratio index
ARFI: acoustic radiation force impulse
AUROC: area under the receiver operating characteristic
CE‐EUS: contrast‐enhanced endoscopic ultrasound
CE‐US: contrast‐enhanced ultrasound
CI: confidence interval
CSPH: clinical significative portal hypertension
CT: computed tomography
OCV: oesophageal collateral veins
OGDS: oesophagogastroduodenoscopy
EUS: endoscopic ultrasound
GLP‐1: glucagon‐like peptide‐1
HCC: hepatocellular carcinoma
HVAT: hepatic vein arrival time
HVPG: hepatic venous pressure gradient
IGV: isolated gastric vein
INR: international normalised ratio
IQR: Interquartile range
LGV: left gastric vein
MASLD: metabolic dysfunction‐associated steatotic liver disease
MELD: model for end‐stage liver disease
MRI: magnetic resonance imaging
NAFLD: non‐alcoholic fatty liver disease
NASH: nonalcoholic steatohepatitis
NITs: non‐invasive tests
NPV: negative predictive value
PH: portal hypertension
PHG: portal hypertensive gastropathy
PPG: portosystemic pressure gradient
PPV: positive predictive value
SWE: shear wave‐based elastography
TD: thoracic duct
TE: transient elastography
TIPS: transjugular intrahepatic portosystemic shunt
US: ultrasound

**Summary**

- EUS technological advancements enhance the detection of portal hypertension‐related signs, matching or surpassing conventional endoscopy, and aiding diagnosis and treatment monitoring.
- EUS‐SWE offers minimally invasive liver fibrosis assessment and treatment monitoring.
- EUS‐PPG measurement provides a minimally invasive method for portal pressure assessment, promising comprehensive evaluation and biopsy in one procedure.
- EUS enables a comprehensive, multiparametric evaluation of portal hypertension within a single session, optimising the diagnostic process and potentially improving patient outcomes.

## 1 Introduction

Portal hypertension (PH) is a complex and potentially life‐threatening condition that results from increased resistance to blood flow within the portal venous system. The prevalence of PH in patients affected by chronic liver disease varies depending on the stage and aetiology of the hepatic condition. For example, studies have reported that PH can be present in approximately 30% of individuals with non‐cirrhotic non‐alcoholic fatty liver disease (NAFLD) [1]—recently renamed as metabolic dysfunction‐associated steatotic liver disease (MASLD) [2]—to 90% in cirrhotic patients [3, 4], and might lead to the development of serious complications such as varices, ascites and hepatic encephalopathy. The diagnosis and management of PH require a multidisciplinary approach that involves a combination of clinical assessment, laboratory tests, imaging studies and invasive procedures [5].

Endoscopic ultrasound (EUS) is a diagnostic modality that has been increasingly used in the evaluation of PH. EUS ++was first introduced in the 1980s as a minimally invasive imaging technique that uses high‐frequency sound waves to produce detailed images of the gastrointestinal tract and surrounding structures [6]. Over the years, EUS has evolved to become an important tool for diagnosing and treating various gastrointestinal conditions, including hepato‐biliopancreatic diseases.

EUS offers several advantages over other imaging modalities in the evaluation of PH. It allows for detailed visualisation of the portal vein system, including the portal, splenic, and mesenteric veins. EUS also provides accurate measurements of the diameter and velocity of blood flow in these vessels, which can help assess the severity of PH and predict the risk of PH‐related complications. In recent years, several studies have investigated the role of EUS in the assessment of PH, including its ability to predict the presence and severity of gastroesophageal varices. EUS can detect small varices that may not be visible on conventional endoscopy and can also provide information on both morphological features and blood flow of the varices, eventually guiding their management [7].

Furthermore, EUS has the potential to play a role in the prevention and early detection of PH. Indeed, it can be used to evaluate the liver parenchyma, detect focal hepatic lesions, and assess the portal vein for any abnormalities, such as thrombosis or stenosis that may lead to PH [8].

This review aims at summarising the current state of knowledge regarding the use of EUS in the evaluation of PH, including its role in predicting the presence and severity of varices and in guiding potential therapeutic interventions. We will also discuss the limitations and future directions of EUS in the evaluation of PH. We present this article in accordance with the narrative review reporting checklist (please, see Supporting Informations).

## 2 Methods

A literature search was conducted using the databases PubMed and Google Scholar. The following MeSH terms—alone or in combination—were adopted: “endoscopic ultrasound”, “portal hypertension”, “liver AND fatty”, “steatohepatitis”, “liver steatosis”, “portal system”, “hepatic cirrhosis”, “liver AND fibrosis”, “endosonography” and “ultrasonic endoscopy”. Additional papers were found by searching the reference list of pertinent articles and on the basis of personal knowledge of the Autho . The last search was performed on 31 May 2024. Only full‐length papers and abstracts written in the English language were considered.

## 3 Key Content and Findings

### 3.1 EUS in the Identification of Collateral Vessels

The presence of portosystemic collaterals (e.g., left gastric, oesophageal, paraesophageal, perisplenic, retrogastric, retroperitoneal, paravertebral, paraumbilical mesenteric and rectal), and hepatofugal blood flow in the portal venous system is a pathognomonic sign of PH [9].

In the past, the ability of EUS to detect gastroesophageal varices was considered inferior to esophagogastroduodenoscopy (OGDS) with several studies reporting that EUS was less accurate, and its sensitivity was largely dependent on the size and grade of varices [10, 11]. However, thanks to the latest equipment's technological advancement of EUS (new‐generation, high‐resolution video‐echoendoscope with miniature probes exerting less pressure on varices), the scenario has evolved [12].

**FIGURE 1:**
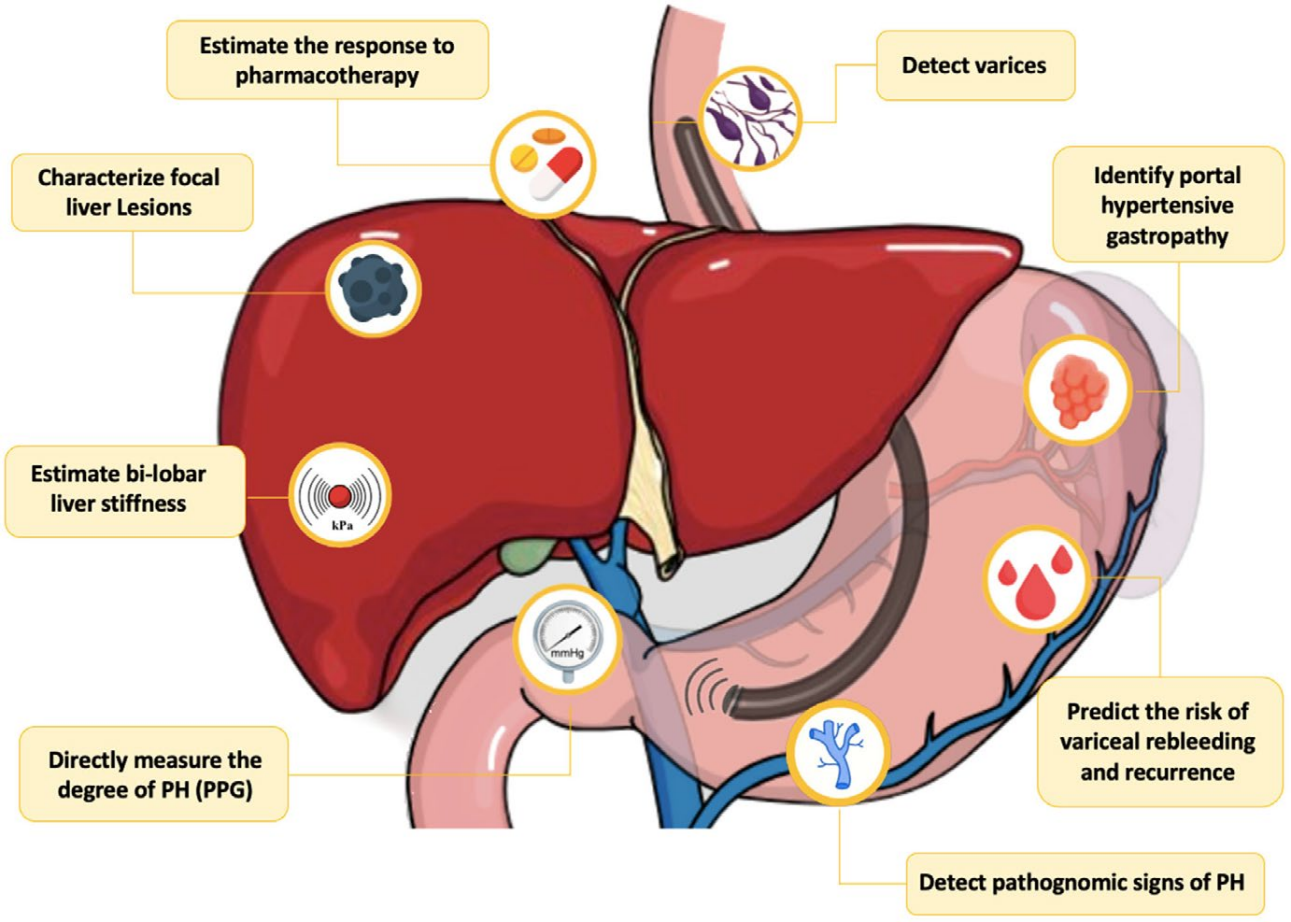
Graphical abstract on the role of endoscopic ultrasound in the assessment of portal hypertension. PH, portal hypertension; PPG, portal pressure gradient.

**FIGURE 2:**
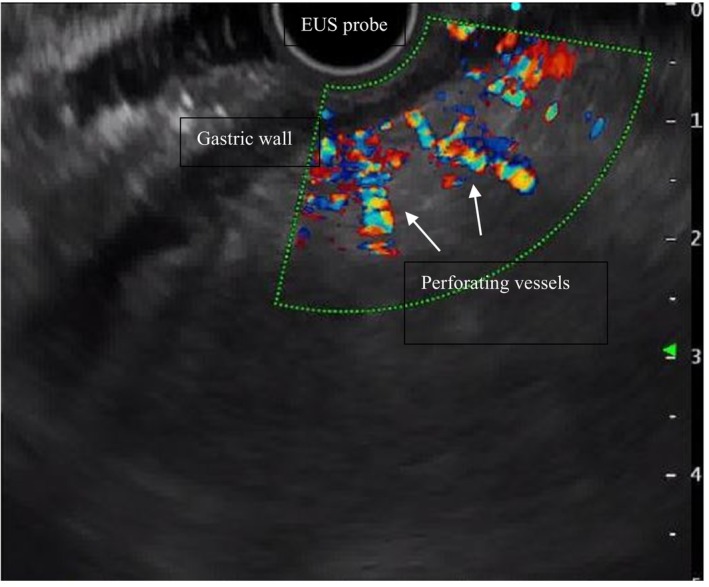
Evaluation of perforating vessels of the gastric wall on colour Doppler during endoscopic ultrasound in a patient with liver cirrhosis and portal hypertension.

Recent evidence has shown that EUS is comparable to conventional OGDS in detecting oesophageal varices [13, 14] and even superior in detecting gastric varices [15, 16] (Figures 1 and 2). In a prospective study by Faigel et al. including 66 cirrhotic patients consecutively undergoing EUS and OGDS, EUS detected oesophageal varices in 48 (72%) patients, compared to 49 (79%) detected by OGDS [13]. These data have been confirmed in a similar prospective study enrolling 52 consecutive patients with hepatic cirrhosis undergoing both OGDS and EUS performed by blinded operators [14]. EUS identified oesophageal varices by the endoscopic vision in 53.8% of included patients with a good correlation with OGDS (*r* = 0.855, *p* < 0.001) [14]. In line with a previous study conducted by Chouduri et al. [15], Lee et al. demonstrated that EUS can detect gastric varices almost twice more often when compared with standard endoscopy (*p* < 0.001) and allows to differentiate gastric varices from thickened gastric folds and other submucosal lesions [14]. In addition, EUS can easily measure the size of gastric varices, which was proven to strongly correlate with their flow volume (*rs* = 0.85, *p* < 0.01) [16].

The use of ultrasound miniature probes contributes to better evaluating intramural and extramural parts of the oesophagus. This EUS‐related characteristic overcomes the limitations of traditional endoscopy's superficial view. Specifically, two types of collateral oesophageal veins can be observed by EUS: periesophageal collateral veins (peri‐OCVs) and paraesophageal collateral veins (para‐OCVs), respectively, located inside and outside the oesophageal wall. The dilation of these vessels is thought to develop as a direct result of PH [14].

This was demonstrated in a prospective study in which 52 cirrhotic patients and 116 dyspeptic patients with no history of liver disease underwent EUS evaluation. The evidence of collateral oesophageal veins was assessed in 48 out of 52 (92.3%) cirrhotic patients, and in 9 out of 116 (0.8%) control subjects, with a statistically significant difference between the two groups (*p* < 0.001). When the described venous abnormalities were used to diagnose the presence of PH, the sensitivity, specificity, PPV and NPV were 92.3%, 94.6%, 84.2% and 97.5%, respectively. Furthermore, the sensitivity, specificity, PPV and NPV of diagnosing PH by the presence of varices were 57.7%, 100%, 100% and 88.3% [14].

The ability to distinguish peri‐OCVs and para‐OCVs using EUS has significant implications for managing PH. Peri‐OCVs are small‐diameter vessels adjacent to the oesophageal adventitia, while para‐OCVs are larger vessels situated further from the oesophageal wall. Identifying these vessels aids in accurately stratifying the risk of variceal recurrence following endoscopic eradication. Clinically, identifying peri‐OCVs and para‐OCVs enables a more tailored approach to patient management. Peri‐OCVs often appear as small, shaggy vessels on EUS, indicating a higher likelihood of variceal recurrence if numerous or large. Para‐OCVs, visualised as distinct, larger vessels, may communicate with oesophageal varices via perforating veins, contributing to recurrence and bleeding risk [17]. Understanding these haemodynamic patterns helps clinicians predict therapeutic response and recurrence risk, facilitating more effective surveillance and treatment plans. EUS provides detailed imaging of these collateral pathways and allows for monitoring treatment efficacy by evaluating changes in vascular structures post‐intervention. Thus, routine use of EUS for assessing peri‐ and para‐OCVs enhances patient management with PH, improving outcomes through precise and individualised care strategies.

Collecting information on portosystemic collateral vessels can be beneficial for monitoring treatment response and predicting variceal rebleeding and recurrence. OCVs often communicate with oesophageal varices via perforating vessels and serve as feeding pathways for varices [18, 19, 20]. This explains the reason why para‐OCV persistence after endoscopic variceal treatment hugely increases the risk of rebleeding and recurrence. In a prospective study including 30 patients receiving endoscopic variceal ligation, a gastric cardia perforating vein diameter greater than 3 mm was associated with a higher likelihood of recurrence of oesophageal varices in 3 months (90.9% vs. 21.0%, *p* < 0.01) [21]. Similarly, in another prospective study examining EUS features before and after band ligation for a first episode of oesophageal variceal bleeding, the presence of para‐OCV larger than 4 mm after band ligation was shown to predict variceal recurrence in 1 year with a sensitivity and specificity of 70.6% and 84.6%, respectively [22].

The main feeding vessel of peri‐ and para‐OCV is the left gastric vein (LGV). Toyonaga et al. demonstrated that a dilated LGV running up the oesophagus was more prevalent in patients with “resistant” varices—that is, those that do not respond adequately to sclerotherapy treatment—when compared with those with non‐resistant varices (100% vs. 3%, respectively; *p* < 0.01). Also, the diameter of the LGV was larger in patients with resistant varices when compared with those with non‐resistant varices (12.4 ± 2.0 vs. 7.8 ± 2.3 mm, respectively; *p* < 0.01) [23]. Supported by such studies, LGV dilation and flow velocity were further evaluated with EUS to assess their predictive role in the recurrence of oesophageal varices. Kuramochi et al. found that both rapid hepatofugal LGV flow and anterior branch dominant LGV pattern appear to be associated with increased odds of post‐eradication oesophageal varices recurrence in one year [24]. In this way, EUS might help to assess the efficacy of the therapeutic management.

In patients with PH, LGV and OCVs are the main ‘inflow’ pathways for gastroesophageal varices, whereas the main ‘outflow’ pathway is the azygos vein, which collects blood from the portal system and feeds it into the systemic circulation. Further EUS studies of the azygos vein evaluating both the diameter and flow using Doppler techniques are needed to evaluate the response to pharmacotherapy administered to decrease blood flow. In an abstract by Sung et al. basal azygos blood flow showed a positive association with the Child‐Pugh grade of cirrhosis (*p* < 0.005). After a bolus injection of terlipressin and somatostatin, a marked decrease in the azygos blood flow was reported after 1 min (24% and 37%), after 5 min (42% and 19%) and after 10 min (40% both). The control group (who received saline solution) showed no significant change in azygos blood flow [25].

In addition to evaluating the azygos vein, it is also important to consider the role of collateral vessels such as the thoracic duct in the context of PH. The thoracic duct, which collects lymph from the lower body and the left side of the upper body, can become engorged and contribute to the collateral circulation seen in PH. Studies have shown that the thoracic duct can show significant changes in diameter and flow characteristics in patients with PH before and after transjugular intrahepatic portosystemic shunt (TIPS) placement. Before TIPS, increased lymphatic flow and dilation of the thoracic duct can be observed, reflecting the heightened collateral circulation due to elevated portal pressures. After TIPS, the reduction in portal pressure often leads to a decrease in the engorgement and flow of the thoracic duct, indicating an improvement in the overall haemodynamic status. EUS could play a crucial role in assessing the response to TIPS by potentially providing detailed imaging of the collateral vessels and changes in haemodynamics. Post‐TIPS, EUS might monitor the reduction in collateral vessel dilation and flow, including the thoracic duct and azygos vein, which could help evaluate the effectiveness of the shunt in alleviating PH [11, 26]. Regular EUS assessments could thus offer valuable insights into how well TIPS is managing portal pressures and might guide adjustments in therapeutic strategies [27].

The thoracic duct also likely plays a role in the pathogenesis of PH‐related complications, such as the development of ascites and oedema. A prospective study by Parasher et al. highlighted that the thoracic duct was dilated in cirrhotic patients with oesophageal varices and ascites, but not in other cirrhotic groups without these conditions [26]. Although the median thoracic duct diameter in 33 portal hypertensive and/or cirrhotic patients was significantly higher compared to control subjects (*p* = 0.003), statistically significant differences between subgroups were not identified. Nonetheless, EUS may be a useful tool for predicting ascites development by evaluating the thoracic duct. For example, a dilated duct might indicate high flow into the lymphatic vascular system, thus serving as a predictive parameter for ascites development (Table 1).

**TABLE 1:** EUS evaluation of the portosystemic vascular system in patients with portal hypertension: relevant published studies.

Author, year (country)	Study design	Cohort	Number of patients	Oesophageal varices (EV)	Gastric varices (GV)	Oesophageal collateral vessels (OCV) and perforating vessels (PV)	Left gastric vein (LGV)	Azygos vein (AV)	Thoracic duct (TD)
Caletti, 1990 (Italy) [10]	OP	Cirrhotic pts with PH	40	EUS was inferior to OGDS in detecting and grading EV (50% vs. 100%, *p* < 0.0005)	EUS was superior to standard endoscopy in detecting GV (55% vs. 25%, *p* < 0.0005)	The diameter of peri‐OCV by EUS was correlated with the endoscopic grade of EV	NA	The diameter of the AV by EUS correlated with PH (*p* < 0.001) and with the endoscopic grade of EV (grade 1: *p* < 0.02; grade 2: *p* < 0.01)	NA
Burtin, 1996 (France) [11]	OP	Cirrhotic pts with PH	58	EUS was inferior to OGDS in detecting EV (55% vs. 88%, *p* < 0.01)	EUS was superior to OGDS in detecting GV (41% vs. 17%, *p* < 0.01)	The detection of PV demonstrated a significant correlation with the presence of GV (*p* < 0.001)	NA	NA	NA
Choudhuri, 1996 (India [15])	OP	Cirrhotic pts with PH	50	EV were detected by EUS in 100% pts with large varices, but only in 45% of small varices	GV were detected more often by EUS (66%) compared with OGDS (34%), *p* < 0.005	The number and size of para‐OCV correlated with the grade of EV (*p* < 0.0005 and *p* < 0.00001, respectively)	NA	NA	NA
Toyonaga, 1996 (Japan) [23]	OP	Pts with EV who underwent sclerotherapy	97	NA	NA	NA	The prevalence and diameter of LGV correlated with sclerotherapy resistant varices (both *p* < 0.01)	NA	NA
Parasher, 1998 (USA) [26]	OP	Pts with PH (29 cirrhotic, 4 non cirrhotic)	33	NA	NA	NA	NA	NA	TD dilatation was detected only in pts with ascites or EV
Lee, 1999 (China) [28]	OP	Cirrhotic pts with PH	18	NA	NA	NA	NA	The administration of vasoactive agents reduced blood flow in the AV	NA
Faigel, 2000 (USA) [13]	OP	Cirrhotic pts with PH (31 with prior haemorrhage)	66	EUS was inferior to OGDS in detecting EV (72% vs. 79%, *p* < 0.0001)	EUS detected gastric varices in 3% pts, compared to 50% detected by OGDS (*p* < 0.0001)	Para‐OCV were predictor of cirrhosis. SE: 97%. SP: 97%	NA	AV diameter was greater for cirrhotic than control pts (*p* < 0.001)	TD diameter was greater for cirrhotic than control pts (*p* < 0.001)
Lee, 2002 (China) [14]	OP	Cirrhotic pts with PH	52	EUS identified EV by the endoscopic vision with a SE of 96.4% and a SP 95.8% (using OGDS as the gold standard)	OGDS detected GV with a SE of 43.8% and a SP 94.4% (using EUS as the gold standard)	The diameter of para‐OCV and peri‐OCV was correlated with Child‐Pugh grading of cirrhosis (*p* = 0.03 and *p* < 0.001, respectively) and grade of EV (both *p* < 0.001) The number of PV was positively correlated with CP grade of cirrhosis (*p* = 0.01) and grade of EV (*p* < 0.001) and GV (*p* < 0.001)	NA	NA	NA
Konishi, 2002 (Japan) [21]	OP	Pts with high‐risk EV	30	NA	EUS detected cardial GV in 100% pts, compared to 70% detected by OGDS	The size of PV (diameter ≥ 3 mm) predicted variceal recurrence in 3 months (90.9% vs. 21.0%, *p* < 0.01)	NA	NA	NA
Kuramochi, 2007 (Japan) [24]	OP	Pts with prior treatment for EV	68	NA	NA	NA	Both rapid hepatofugal LGV flow (> 12 cm/s) and anterior branch dominant LGV pattern predicted post‐eradication EV recurrence (*p* = 0.0044)	NA	NA
Imamura, 2014 (Japan) [16]	OP	Pts with GV	24	NA	The size of GV by EUS correlated with their flow volume (*rs* = 0.85, *p* < 0.01)	NA	NA	NA	NA
Carneiro, 2016 (Brazil) [22]	OP	Pts with EV who underwent endoscopic band ligation	30	NA	NA	The size of para‐OCV predicted variceal recurrence in 1 year after (SE: 70.6%. SP: 84.6%) and before (SE: 52.9%. SP: 96.3%) band ligation	NA	NA	NA

Abbreviations: AV, azygos vein; EUS, endoscopic ultrasound; EV, oesophageal varices; GV, gastric varices; LGV, left gastric vein; NA, not assessed; OP, observational prospective; OCV, oesophageal collateral vessels; OGDS, esophagogastroduodenoscopy; PH, portal hypertension; pts, patients; PV, perforating vessels; SE, sensitivity; SP, specificity; TD, thoracic duct.

### 3.2 Contrast‐Enhanced EUS

Contrast‐enhanced (CE)‐EUS is a technology that involves the use of microbubbles as contrast agents, which are injected into the bloodstream to improve visualisation of blood vessels and surrounding tissues. This approach builds upon the principles of transabdominal ultrasound, where similar contrast‐enhancement techniques are employed to improve imaging of internal structures. Currently, the main clinical use of CE‐EUS is the investigation of pancreatic lesions, abnormalities of the biliary tract, submucosal tumours, and supra‐ or sub‐diaphragmatic lymph nodes.

A growing number of authors have recently explored the potential of CE‐EUS in assessing PH and its complications. Two papers published by the same study group, respectively a retrospective study involving 62 cirrhotic patients, and a prospective study enrolling 29 cirrhotic patients [29, 30], reported that CE‐EUS improved the quality of the assessment of varices when compared with standard colour Doppler‐EUS. Specifically, the detecting rate of perforating veins rose from 77.4% to 96.8% and 31.0% to 75.9%, respectively [29, 30].

A recent study evaluated the role of CE‐EUS in the assessment of portal hypertensive gastropathy (PHG). A total of 20 patients with compensated liver cirrhosis were enrolled. Among them, 10 patients with endoscopic PHG exhibited contrast enhancement on EUS. On the other hand, one of 10 patients without endoscopic evidence of PHG showed enhancement during EUS (*p* = 0.0001) [31]. Despite the limitations associated with the very small sample size, this study suggests that CE‐EUS may be able to identify PHG earlier and with greater sensitivity than conventional OGDS, which is currently the gold‐standard diagnostic tool. However, further studies are needed to support this assumption [31].

In addition, CE‐EUS could be able to investigate intrahepatic haemodynamic changes caused by PH, by assessing parameters such as hepatic vein arrival time (HVAT). HVAT is the time (s) taken for the microbubble contrast agent to arrive at the hepatic vein after injection. A demonstrated inverse relationship between HVAT and the severity of liver histological grade and PH has been shown in CE‐US, suggesting that HVAT is useful for the non‐invasive clinical significative portal hypertension (CSPH) prediction in patients with compensated cirrhosis [32]. At present, the scientific evidence supporting the measurement of HVAT in CE‐EUS is currently insufficient, creating a research gap that awaits exploration. Indeed, the measurement of HVAT in CE‐EUS lacks advantages over CE‐US in single‐parameter assessment due to its invasiveness; however, it holds potential as valuable information to be integrated into a multiparametric EUS assessment (Table 2).

**TABLE 2:** Contrast‐enhanced EUS for the assessment of portal hypertension: Relevant published studies.

Author, year (country)	Study design	Number of patients	Signs of PH within CE‐EUS
Sato et al., 2003 (Japan) [29]	Retrospective cohort study	62	The sensitivity of EUS for the detection of PV raises from 77.4% to 96.8% using contrast enhancement
Sato et al., 2004 (Japan) [30]	Prospective cohort study	29	The SE of EUS for the detection of PV raises from 31.0% to 75.9% using contrast enhancement
Macedo, 2016 (Brazil) [33]	Retrospective cohort study	20	One of 10 patients without OGDS evidence of PHG showed enhancement during EUS (*p* = 0.0001), suggesting that CE‐EUS may be able to identify PHG earlier and with greater sensitivity than conventional OGDS

Abbreviations: CE‐EUS, contrast‐enhanced endoscopic ultrasound; EUS, endoscopic ultrasound; OGDS, esophagogastroduodenoscopy; PH, portal hypertension; PHG, portal hypertensive gastropathy, PV, perforating vessels; SE, sensitivity.

### 3.3 EUS‐Guided Shear‐Wave Elastography

The field of minimally or non‐invasive liver disease assessment is expanding rapidly. While liver biopsy remains the gold standard for fibrosis assessment, it is not suitable for serial monitoring due to its invasive nature and potential for serious complications, such as haemorrhage, transient bacteraemia, bile peritonitis and other incidental organ injuries. Additionally, liver biopsy only samples a small portion of the liver, leading to sampling variability [34].

Transabdominal shear wave‐based elastography (SWE) is a popular non‐invasive method for evaluating liver fibrosis and PH‐related signs [5]. SWE measures shear wave velocity, which correlates with tissue elasticity. It includes transient elastography (TE) and acoustic radiation force impulse (ARFI) techniques. TE, widely used and validated, is easy, fast, cost‐effective and repeatable [5].

ARFI techniques, including point shear wave elastography (p‐SWE) and 2D shear wave elastography (2D‐SWE), offer the advantage of B‐mode visualisation and do not require specialised devices. They are potentially better suited for patients with chronic liver disease, allowing for bilobar assessment of hepatic parenchyma. This is significant as liver stiffness can vary between lobes, as shown in a study where stiffness was higher in the left lobe compared to the right (17.9 vs. 14.9 kPa; *p* = 0.019) [35].

Recent advances in non‐invasive tests (NITs) have significantly evolved their role from being primarily diagnostic to serving increasingly important prognostic functions. Tools such as SWE and MELD (model for end‐stage liver disease) have become integral components when used in combination for prognostic assessment in liver disease. The Baveno VII guidelines have introduced a “rule of 5” (10–15–20‐25 kPa) for liver stiffness measurement using transabdominal TE, establishing specific stiffness thresholds that correlate with varying levels of risk for hepatic decompensation and liver‐related mortality [5].

By combining SWE results with the MELD score, a comprehensive risk profile can be developed. This integrated approach helps to stratify patients and guide management decisions:

- Mild fibrosis (SWE < 10 kPa) and Low MELD (< 10): Low risk of complications.
- Moderate fibrosis (10–15 kPa) and Moderate MELD (10–19): Intermediate risk, requiring regular monitoring.
- Advanced fibrosis (> 15 kPa) and High MELD (≥ 20): High risk, necessitating aggressive therapeutic interventions and potential consideration for liver transplantation.

The integration of SWE and the MELD thresholds into clinical practice allows for better risk assessment and management planning.

Nevertheless, despite their undoubtable utility, transabdominal ultrasound approaches face limitations. VCTE (vibration‐controlled TE) struggles with a low overall positive predictive value and suboptimal performance in 30% of morbidly obese patients. It is also restricted to assessing only the right liver lobe, which can affect accuracy in certain cases [5, 36, 37, 38].

**FIGURE 3:**
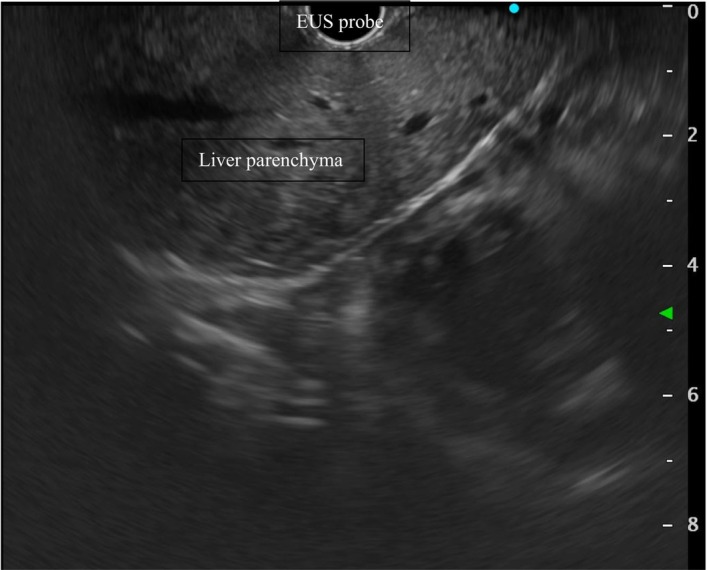
Endoscopic ultrasound image of the hepatic left lobe in liver cirrhosis. The image is obtained from the cardial region.

Since the aforementioned methods can be applied in EUS, EUS‐guided elastography can easily overcome these technical issues, providing a higher degree of sensitivity [39], as the signal only has to pass through the thin gastric wall, without any further anatomical impediments [40] (Figure 3).

In a recent retrospective pilot study by Choi et al., 27 patients with chronic liver disease of non‐specified aetiology underwent the same endoscopic session EUS‐SWE, EUS‐guided liver biopsy, and EUS‐PPG [41]. Left hepatic lobe EUS‐SWE ≥ 12.5 kPa had the highest combined sensitivity and specificity (85.7% and 80.0%) in predicting histological stage ≥ 3 fibrosis, compared with the right hepatic lobe EUS‐SWE ≥ 12.5 kPa (83.3% and 69.0%). Furthermore, EUS‐SWE left lobe ≥ 12.5 kPa was significantly associated with fibrosis stage ≥ 3 on liver biopsy (85.7% vs. 20% in case of SWE < 12.5 kPa, *p* = 0.001), clinical cirrhosis (78.6% vs. 30%, *p* = 0.017), INR ≥ 1.05 (61.5% vs. 20%, *p* = 0.046), gastro‐oesophageal varices (53.8% vs. 10%, *p* = 0.029) on OGDS, Fibrosis‐4 (Fib‐4) score > 3.25 (62.5% vs. 10%, *p* = 0.066), AST to platelet ratio index (APRI) score > 2 (62.5% vs. 10%, *p* = 0.019). No complications were associated with EUS‐SWE, supporting the feasibility and safety of the procedure [42].

To address the correlation between EUS‐SWE and percutaneous SWE‐TE, a pilot study by Kohli et al. provides valuable insights [42]. This single‐centre, prospective, nonrandomised tandem study compared the diagnostic accuracy of EUS‐SWE and VCTE in 42 patients undergoing liver biopsy due to unreliable NITs. The study evaluated EUS‐SWE in both the left and right lobes and compared its performance with VCTE. The results showed that the cross‐validated AUROCs for advanced fibrosis were comparable between VCTE and EUS‐SWE, with VCTE having an AUROC of 0.87 (95% CI, 0.76–0.97), EUS‐SWE for the left lobe at 0.80 (95% CI, 0.64–0.96) and for the right lobe at 0.78 (95% CI, 0.62–0.95). For cirrhosis, VCTE had an AUROC of 0.90 (95% CI, 0.83–0.97), while EUS‐SWE for the left lobe was 0.96 (95% CI, 0.90–1.00) and for the right lobe was 0.90 (95% CI, 0.80–1.00). Importantly, VCTE was unreliable in 8 patients who were successfully assessed by EUS‐SWE.

These findings indicate that EUS‐SWE has diagnostic accuracy comparable to VCTE for assessing liver fibrosis and cirrhosis, making it a reliable alternative, particularly in patients where VCTE may be limited [42].

More clinical studies are underway to correlate and validate EUS‐SWE values with non‐invasive fibrosis scoring systems and liver biopsy (ClinicalTrials.gov Identifier: NCT04115046, NCT05728697, NCT05097963) but also to evaluate the accuracy of EUS‐SWE to assess PH in patients with liver cirrhosis (NCT03155282).

Furthermore, a recent abstract illustrated the utility of EUS‐SWE in predicting various signs of decompensated cirrhosis (i.e., gastroesophageal varices and hepatic encephalopathy) and in monitoring the response to drugs such as non‐selective beta‐blockers and glucagon‐like peptide‐1 (GLP‐1) agonists, opening the door to future studies with larger sample sizes [43].

In conclusion, since EUS‐SWE can survey and quantify the entire hepatic region of interest, this tool might play a key role in the diagnostic work‐up of liver fibrosis by both the optimisation of biopsy indications and the reduction of pointless samples. Moreover, EUS‐SWE can be employed for the serial monitoring of dynamic changes in hepatic fibrosis in patients receiving surveillance endoscopy for gastroesophageal varices.

Further scientific investigations would be useful to confirm the non‐inferiority of EUS‐SWE as a diagnostic tool in patients with chronic liver disease when compared to transabdominal SWE [44] (Table 3).

**TABLE 3:** EUS‐guided elastography in liver disease: Relevant published studies.

Author, year (country)	Study design	Cohort	Number of patients	Results
Schulman, 2018 (USA) [39]	Pilot prospective cohort study	Pts undergoing EUS for any indication	50	Increased LFI scores in cirrhotic group when compared to fatty liver (3.2 vs. 1.7, *p* < 0.001) and normal (3.2 vs. 0.8, *p* < 0.001) groups
Choi, 2021 (USA) [41]	Pilot retrospective cohort study	Pts with various chronic liver diseases	27	EUS‐SWE of the left hepatic lobe ≥ 12.5 kPa: greater SE and SP (85.7%, 80%) in predicting histological stage ≥ 3 fibrosis, when compared to EUS‐SWE of the right lobe, clinical cirrhosis, INR ≥ 1.05, EV/GV +, APRI score > 2, and Fib‐4 > 3.25
Kohli, 2022 (USA) [42]	Prospective, non‐randomised tandem study	Pts with undergoing liver biopsy sampling, VCTE and EUS‐SWE because of unreliable noninvasive testing	42	Acceptable SE and SP for EUS‐SWE of the liver in assessing significant and advanced fibrosis; no statistically significant difference was found in the AUROCs for EUS‐SWE and VCTE
Ayele, 2023 (USA) [43]	Retrospective cohort study	Pts with chronic liver disease undergoing endo‐hepatologic workup	62	Hepatic EUS‐SWE measurements could predict multiple parameters of decompensated cirrhosis (varices, HE), and successfully monitor the pharmacological response

Abbreviations: APRI, AST to platelet ratio index; EUS, endoscopic ultrasound; EV, oesophageal varices; GV, gastric varices; HE, hepatic encephalopathy; LFI, liver fibrosis index; NA, not assessed, PH, portal hypertension; pts, patients; RTE, real‐time elastography; SE, sensitivity; SP, specificity; SWE, shear‐wave elastography; VCTE, vibration‐controlled transient elastography.

### 3.4 EUS‐Guided Portal Pressure Gradient Measurement

In recent years, the importance of EUS in the management of PH has exponentially grown due to the possibility of measuring the portosystemic pressure gradient (PPG). The PPG accurately reflects the degree of PH, and it is one of the most accurate prognostic indicators of liver disease [32].

Despite the increasing use of NITs for assessing patients with chronic liver disease, the hepatic venous pressure gradient (HVPG) remains the gold standard measurement of PPG [5]. HVPG is measured by inserting a catheter into the hepatic veins via a transjugular approach during interventional radiology and calculating the pressure difference between the hepatic vein and the wedged hepatic vein. This gradient reflects the resistance to blood flow within the liver, providing indirect insight into the severity of PH. The normal value of HVPG is between 1 and 5 mmHg; a pressure gradient above > 5 mmHg indicates PH. Patients have a negligible risk of developing PH‐related complications of advanced chronic liver disease as long as HVPG is < 10 mmHg. Contrarily, the risk of developing varices, variceal bleeding, ascites, spontaneous bacterial peritonitis, hepato‐renal syndrome and hepatic encephalopathy exponentially increases in subjects with HVPG ≥ 10 mmHg, which defines CSPH [45]. HVPG monitoring is crucial for stratifying patient prognosis, evaluating the progression of advanced chronic liver disease and assessing the success of therapeutic or preventive interventions.

EUS‐guided portal pressure gradient measurement (EUS‐PPG) has been recently introduced as an advanced diagnostic technique designed to measure directly portal vein pressure through endoscopic ultrasound guidance. The apparatus for PPG measurement includes a 25‐gauge fine‐needle aspiration needle and a compact manometer with non‐compressible tubing [46]. During the procedure, the 25‐gauge needle is directly inserted into the target vessels, and three separate measurements are taken using the attached pressure transducer. These measurements are then averaged to determine the PPG.

After a few animal studies that ascertained the feasibility, safety and accuracy of EUS‐PPG, its first application on a human subject with a 22‐gauge needle was documented in 2014, confirming the results of a previous session of interventional radiology measurement [47]. In 2017, Huang et al. published the first observational cohort study on EUS‐PPG including 28 patients with a history of liver disease or suspected hepatic cirrhosis [48]. PPG measurements went from 1.5 to 19 mmHg and had a great correlation with clinical parameters of PH such as the presence of varices (*p* = 0.0002), PH‐related gastropathy (*p* = 0.007) and thrombocytopenia (*p* = 0.036). PPG was increased in patients with clinically evident cirrhosis (*p* = 0.005). This study showed a 100% technical success rate without intra‐ or post‐procedural adverse events. Despite 4 patients with an INR > 1.2, 16 being thrombocytopenic and 3 with blood urea > 30 mg/dL, no bleeding episodes were reported [48]. An updated abstract from the same researchers with 51 patients undergoing EUS‐PPG, confirmed the preliminary data from the pilot study [49].

A recent prospective study evaluated the consistency between EUS‐PPG and HVPG measurements in nine patients with acute or subacute PH [50]. Subsequent HVPG measurements failed in two patients with Budd‐Chiari syndrome. EUS EUS‐PPG and HVPG average values were 18.07 ± 4.32 mmHg and 18.82 ± 3.43 mmHg, respectively (Pearson's correlation coefficient 0.923, *p* < 0.001). The time to perform either procedure was comparable and there were no adverse events [50].

Further observational studies on EUS‐PPG with limited sample sizes have been published so far [51, 52]. Results from a recent systematic review and meta‐analysis on eight single‐centre studies including 178 patients with liver disease revealed a technical success of 94.6% in achieving PPG measurement (95% confidence interval‐CI 88.5%–97.6%; *I* ^2^ = 0) [53]. The pooled rate of clinical success, defined as either the correlation between the fibrosis stage on histological examination and the PPG measurement, or by the agreement between HVPG and PPG, was 85.4% (95% CI: 51.5%–97.0%; PI = 2%–100%; *I* ^2^ = 70). The authors of the study suggest that this discrepancy may be attributed to variabilities in both the procedural protocol and the technical expertise of the endosonographers involved in doing EUS‐PPG [53]. Moreover, the pooled rate of total adverse events was 10.9% (95% CI 6.5%–17.7%; PI = 5%–23%; *I* ^2^ = 4), though the majority of them (93.7%) were considered mild according to the ASGE lexicon [54]; abdominal pain (11.0%) and bleeding (3.6%) were the more frequent [53].

A great advantage of EUS‐PPG is its multimodal approach, which allows for the simultaneous assessment of portal vein pressure, varices and liver biopsy within a single procedure. This comprehensive capability could facilitate the management of PH by integrating multiple assessments into one session. By consolidating these tasks, EUS‐PPG could reduce the need for multiple separate interventions, potentially minimising patient discomfort and overall procedural time. A recent study conducted in the United States aimed to compare EUS‐PPG and HVPG in terms of costs, procedure times, and patient outcomes [55]. Fifty‐three patients underwent EUS‐guided PPG and liver FNB, while 46 underwent HVPG and transjugular liver biopsy. The technical success rates for EUS‐PPG and HVPG measurements were 100% and 97.8%, respectively, with no post‐procedure adverse events observed in either group. The median radiation dose for the HVPG measurement + transjugular liver biopsy group was 118.35 mGy (IQR 65–241 mGy), which is equivalent to the radiation exposure from performing 10 abdominal CT scans. In comparison, EUS‐guided PPG and FNB demonstrated a lower median economic burden, with a significant difference of $6326 (*p* < 0.0001), as well as a shorter median procedure time of 17 min (*p* < 0.0001), while anaesthesia time remained similar (*p* = 0.84) [55].

In patients with specific chronic liver disease aetiologies, HVPG may not always be reliable or technically feasible in portal pressure measurement. For instance, the presence of steatosis and fibrotic changes in MASLD can lead to discrepancies in pressure measurements due to non‐uniform liver damage and extrahepatic factors affecting portal pressure [56]. Similarly, in primary biliary cholangitis (PBC), multiple studies have shown that PH in early PBC is often presinusoidal, and early bile duct damage and fibrosis may not significantly alter HVPG until advanced stages of the disease [57]. HVPG also faces challenges in cases of portal vein thrombosis, as the presence of the thrombus disrupts normal blood flow and pressure dynamics, leading to inaccurate or misleading measurements.

EUS‐PPG addresses these limitations by providing direct measurements of portal vein pressure under US guidance, thus offering a more precise assessment of PH in particular settings. It can be particularly beneficial in patients with MASLD and presinusoidal diseases, where HVPG may not fully capture the extent of portal pressure changes [58].

However, for patients at a higher risk of bleeding, specifically, those for whom HVPG is favoured over percutaneous biopsy, it remains plausible that HVPG offers superior safety compared to EUS‐PPG. Furthermore, if we were to regard EUS‐FNB as a stand‐alone procedure, it might prove less advantageous in terms of cost‐effectiveness than transabdominal liver biopsy.

These potential benefits of EUS‐PPG must be pondered against the drawback of using deep sedation, which, when necessary to do the endoscopic examination, may impair the accuracy and repeatability of PPG measurement [59]. Deep sedation is known to result in erroneous HVPG assessments [60], particularly for low midazolam dosages, which may not be enough to perform EUS‐PPG. Before making a final determination, it is necessary to further explore the impact of sedation on EUS‐PPG measurement.

In conclusion, although EUS‐PPG might offer several advantages, including its multimodal approach and potential benefits for patients with specific chronic liver diseases where HVPG may be less effective, further research is needed. Randomised controlled trials and additional studies will be crucial in determining the full scope of EUS‐PPG's applicability and its comparative effectiveness with traditional HVPG. These future studies will help clarify EUS‐PPG's role in clinical practice and address any remaining uncertainties regarding its optimal use (Table 4).

**TABLE 4:** EUS‐guided portal pressure gradient measurement: Relevant published studies conducted on humans.

Author, year (country)	Study design	Cohort	Number of patients	Results
Huang, 2017 (USA) [48]	Pilot retrospective cohort study	Pts with liver disease or suspected cirrhosis	28	EUS‐PPG had an adequate correlation with clinical parameters of portal hypertension and clinical signs of cirrhosis (*p* < 0.05). No adverse events
Samarasena, 2018 (USA) [61]	Pilot retrospective cohort study (updated data of Huang et al., 2017 [48])	Pts with liver disease or suspected cirrhosis	51	EUS‐PPG had an adequate correlation with EV and GV, PHG, low platelet count, and cirrhosis. 3 pts complained of mild abdominal pain
Zhang, 2021 (China) [50]	Prospective cohort study	Pts with chronic liver disease undergoing endo‐hepatologic workup	12	Technical success of EUS‐PPG in 11 (91.7%) out of 12 pts. Good correlation between EUS‐PPG and HVPG in 9 pts (*p* < 0.01). No adverse events
Choi, 2022 (USA) [52]	Retrospective cohort study	Pts undergoing EUS‐PPG for chronic liver disease	83	Technical success of 100% for EUS‐PPG. There were no early or late major adverse events
Wang, 2023 (China) [55]	Retrospective comparative study	Pts undergoing EUS‐PPG or HVPG + liver biopsy	99 (53 of EUS‐PPG group, 46 of the HVPG group)	Technical success of 100% for EUS‐PPG, 97.8% for HVPG. No adverse events. EUS‐PPG had a lower median charge and procedure time when compared to HVPG

Abbreviations: EUS, endoscopic ultrasound; EUS‐PPG, EUS‐guided portal pressure; EV, oesophageal varices; GV, gastric varices; HVPG, hepatic venous pressure gradient; PHG, portal hypertension‐related gastropathy.

## 4 Discussion

Over the past years, EUS has emerged as a promising and valuable tool for the evaluation of PH at all stages of its pathophysiological progression, spanning from early to advanced phases.

For example, in decompensated cirrhotic patients with eloquent clinical manifestations of PH, EUS may help estimate the risk of variceal bleeding, treat varices, assess the risk of variceal recurrence after treatment, evaluate the response to drug treatments, detect portosystemic collateral vessels, unearth and investigate malignant lesions of the liver parenchyma [62, 63].

According to the Baveno VII guidelines, a variety of NITs incorporating laboratory markers, hepatic and splenic stiffness measurements, and advanced imaging techniques have emerged as reliable predictors of CSPH in patients with liver disease [5]. The utilisation of these tests aids in determining the optimal timing for OGDS, thereby avoiding unnecessary invasive procedures for patients.

However, in this regard, EUS could turn the tide in the coming years: not only it offers comparable diagnostic information when compared with conventional endoscopy, but also introduces a remarkable opportunity to perform numerous additional diagnostic evaluations within a single session. Indeed, EUS could unleash its full potential, particularly in situations where the prediction of CSPH is challenging. This difficulty arises due to NITs failing to reach established cut‐off values, making clinicians wander in a ‘grey area’. The primary reason for this discrepancy lies in the fact that these NITs were validated in studies that identified HVPG as the gold standard tool for the diagnosis of PH. However, as previously mentioned, in specific liver pathologies like metabolic cirrhosis or porto‐sinusoidal vascular disease, HVPG fails to accurately reflect the true extent of PH.

When compared to the US, EUS can demonstrate non‐inferiority in measuring hepatic and splenic stiffness and can even present a higher accuracy, particularly in obese patients or those with limited intercostal spaces or excessive gas or bloating, and identify early signs of PH, such as para‐OCV, peri‐OCV, perforating vessels and portosystemic collateral vessels, facilitating decision‐making regarding the initiation of non‐selective beta‐blockers therapy. Furthermore, EUS might enable direct measurement of PPG, offering an accurate quantification of the degree of PH.

Contrarily, notable drawbacks accompany EUS in PH management. First, CE‐EUS and EUS‐SWE are emerging but still under‐researched modalities, with small sample sizes limiting the generalisability of findings. Second, we must not overlook, though minimal, the inherent invasiveness of EUS which carries potential complications and anaesthesiologic risks. These risks include adverse reactions to sedation or anaesthesia, respiratory depression and cardiovascular issues, which may be exacerbated in patients with advanced chronic liver disease [64]. To better assess whether EUS is the most appropriate method or if less invasive alternatives should be considered, the use of prognostic scoring systems could help guide the decision‐making process. Another limitation is that the administration of sedative medications during EUS‐PPG can potentially affect the PH assessment. Current expert evidence‐based consensus suggests that HVPG measurement ideally requires minimal to no sedation, with a maximum midazolam dose of 0.02 mg/kg [65]. However, all published EUS‐PPG studies to date have utilised moderate to deep sedation, which introduces significant variability in HVPG readings and may lead to inaccuracies in assessing PH severity [53]. Addressing this challenge is complicated by the unfeasibility of performing EUS under mild sedation. One potential approach could involve conducting dedicated studies to investigate the impact of midazolam on PPG measurement during EUS, to obtain results corrected for the exact dose of midazolam, or exploring alternative sedatives that might exert less influence on vascular resistance and, consequently, on the PPG.

Furthermore, EUS is currently only available at tertiary referral centres and demands extensive training, which remains inadequately defined. The most recent position statements do not address the specific training requirements for Endohepatology, highlighting a gap in comprehensive educational guidelines [66]. Given that Endohepatology is a relatively new and specialised field, effective training must integrate both clinical expertise in hepatology and hands‐on technical skills in EUS. To address this, there should be a well‐defined curriculum that includes rigorous hands‐on practice and theoretical knowledge.

In summary, EUS is a valuable diagnostic modality in the evaluation of PH, with the potential to improve patient outcomes through earlier diagnosis and better risk stratification. Further research is needed to fully explore the capabilities and limitations of EUS in this setting and to determine the optimal use of this technology in the management of PH (Table 5).

**TABLE 5:** The role of endoscopic ultrasound in the assessment of portal hypertension: Summary.

*Standard EUS*
EUS is comparable to OGDS for detecting and grading oesophageal varices but offers superior sensitivity and specificity for gastric varices detection EUS identifies pathognomonic signs of PH more effectively than transabdominal ultrasound and evaluates key vascular structures to assess the risk of variceal rebleeding EUS can be useful for estimating the response to pharmacotherapy for PH and predicting ascites development
*CE‐EUS*
CE‐EUS improves detection of PV and may identify PHG earlier and more sensitively than conventional OGDS CE‐EUS helps characterise focal liver lesions and estimate hepatic vein anatomy to predict clinically CSPH in compensated cirrhosis
*EUS‐SWE*
EUS‐SWE might estimate liver stiffness with superior sensitivity over transient elastography in patients with obesity and ascites and may aid in monitoring drug response
*EUS‐PPG*
EUS‐PPG provides direct estimation of PH degree and may be advantageous in cases with anatomical abnormalities or Budd‐Chiari syndrome and in presinusoidal liver diseases, when compared to HVPG
*EUS‐guided liver biopsy*
EUS can guide biopsies of liver parenchyma or specific focal liver lesions, facilitating accurate sampling, in a single‐session, multimodal approach

Abbreviations: CSPH, clinical significative portal hypertension; CT, computed tomography; EUS, contrast‐enhanced endoscopic ultrasound; EUS, endoscopic ultrasound; HVPG, hepatic venous pressure gradient; OGDS, oesophagogastroduodenoscopy; PH, portal hypertension; PHG, portal hypertensive gastropathy; PV, perforating veins; SWE, shear wave‐based elastography.

## 5 Future Directions

This overview presents EUS as a valuable tool in the assessment of PH, given its wide range of applications, high technical success rate, and minimal complication rate. Its primary advantage is being a ‘one‐stop‐shop’ procedure, allowing for a comprehensive array of tasks. The idea that an endoscopist can diagnose and prognosticate PH without relying on radiology has now been shown to be practical and safe. A key disadvantage is that currently EUS is only accessible in tertiary centres, and its clinical applicability is primarily focused on the gastrointestinal tract, bile ducts, and pancreatic gland. To promote wider adoption of EUS in hepatology, and therefore in PH management, several measures are needed: conducting large‐scale randomised controlled trials to demonstrate EUS's effectiveness compared to standard alternatives before advocating for its inclusion in clinical guidelines; defining training and competency standards for endohepatologists; adopting a multidisciplinary approach involving both endoscopists and hepatologists; addressing implementation challenges in non‐tertiary settings through training and remote support; raising awareness among healthcare providers about the benefits of EUS. These steps would significantly enhance EUS adoption, improving diagnosis, prognosis, and management of PH and its complications.

## Author Contributions

**Fabrizio Termite**, **Federica Borrelli de Andreis** and **Luca Miele:** conception and design. **Antonio Gasbarrini**, **Cristiano Spada** and **Luca Miele:** administrative support. **Fabrizio Termite** and **Federica Borrelli de Andreis:** provision of study materials or patients, collection and assembly of data and data analysis and interpretation. All authors involved in the manuscript writing and final approval of manuscript.

## Conflicts of Interest

The authors declare no conflicts of interest.

## Supporting information


Data S1.

## Data Availability

Data sharing not applicable to this article as no datasets were generated or analysed during the current study.
