# Supplementary material for: The Role of Endoscopic Ultrasound in Assessing Portal Hypertension: A State‐of‐the‐Art Literature Review and Evolving Perspectives
Source: Liver Int. 2024 Nov 27;45(4):e16176. doi: 10.1111/liv.16176 (PMC11927608; doi:10.1111/liv.16176)
Supplement: Supplementary file 1 — Data S1. [file LIV-45-0-s001.docx]

**SUPPLEMENTARY MATERIALS**

| **Items** | **Specification** |
| --- | --- |
| Date of Search (specified to date, month and year) | 31^st^ May, 2024 |
| Databases and other sources searched | PubMed  Google Scholars |
| Search terms used (including MeSH and free text search terms and filters) | “endoscopic ultrasound”, “portal hypertension", “liver AND fatty”, “steatohepatitis”, “liver steatosis”, “portal system”, “hepatic cirrhosis”, “liver AND fibrosis”, “endosonography”, “ultrasonic endoscopy”. |
| Timeframe | January 1990 – May 2024 |
| Inclusion and exclusion criteria (study type, language restrictions etc.) | Original articles, case reports, case series, reviews, and abstracts in English language were included. |
| Selection process (who conducted the selection, whether it was conducted independently, how consensus was obtained, etc.) | Two reviewers (FT and FBdA) performed the literature search and evaluated all the included articles for manual data extraction. Disagreements between reviewers regarding data collection were solved through discussion between the Authors. |
| Any additional considerations, if applicable | Not applicable |

***Table 1.*** *The search strategy summary.*
